# Integration of MRI radiomics features and clinical data for predicting neurological recovery after thoracic spinal stenosis surgery: a machine learning model

**DOI:** 10.3389/fmed.2025.1633633

**Published:** 2025-10-22

**Authors:** Bin Zheng, Zhenqi Zhu, Panfeng Yu, Yan Liang, Haiying Liu

**Affiliations:** Spine Surgery, Peking University People’s Hospital, Beijing, China

**Keywords:** thoracic spinal stenosis, MRI radiomics, machine learning, neurological recovery, predictive

## Abstract

**Background:**

Thoracic spinal stenosis (TSS) is a rare yet debilitating condition, often requiring surgical decompression. Prognostic assessments traditionally rely on single clinical or imaging features, limiting prediction accuracy. This study explores whether radiomics-based models enhance outcome prediction in TSS.

**Methods:**

We retrospectively enrolled 106 surgically treated TSS patients (2012–2022), collecting clinical data and T2 axial MRI scans. Radiomics features were extracted from the most stenotic level, followed by rigorous feature selection (ICC > 0.9, U-test, Spearman, mRMR, and LASSO). Six machine learning classifiers were trained using radiomics and/or clinical data. Model performance was evaluated using AUC on an independent test set.

**Results:**

Radiomics models outperformed clinical models (SVM AUC: 0.824 vs. 0.731). The combined radiomics–clinical model achieved the highest test-set AUC of 0.867, offering improved sensitivity and specificity.

**Conclusion:**

In this preliminary exploratory study, integrating MRI radiomics with clinical data appeared to improve prediction of neurological recovery in TSS. These findings suggest that radiomics may enable objective, high-dimensional assessment of spinal cord pathology and potentially support individualized surgical decision-making, although further validation in larger, multicenter prospective cohorts is required.

## Introduction

Thoracic spinal stenosis (TSS) is a relatively rare cause of spinal cord compression, often resulting from ossification of the ligamentum flavum or the posterior longitudinal ligament in the thoracic spine (1). It frequently leads to thoracic spinal cord dysfunction, and severe cases require surgical decompression (1). Many clinical studies focus on predicting outcomes for TSS or thoracic spinal cord lesions, but most rely on single-factor assessments with limited predictive dimensions (2). With the widespread use of MRI, research attention shifts to T2 intramedullary high signal intensity (ISI) and its quantitative assessment. Kim et al. validate a similar conclusion using the signal intensity ratio (SIR): a lower SIR correlates with a higher postoperative JOA recovery rate, and the preoperative JOA score itself also acts as a positive prognostic indicator (3). Hitchon reports Increased signal intensity on T2-weighted MRI images correlated with lower Frankel and JOA scores compared to those without (4).

These studies establish the foundation for combined imaging–clinical assessments. However, feature dimensionality remains low, largely relying on visually measurable morphological parameters (such as spinal canal diameter or the number of compressed segments) or a single grayscale index. This approach fails to capture the potential textural heterogeneity in the lesion. Additionally, generalizability remains limited—most analyses still use traditional single-factor or multivariate regression methods, lacking comprehensive machine learning frameworks. Nevertheless, previous cutting-edge research in spinal disorders demonstrates radiomics’ potential (5–9). For example, one multicenter study combines MRI radiomics and deep learning features to predict postoperative upper-limb muscle strength recovery in spinal cord injury patients, reporting an AUC near 0.89 on the test set (10).

Researchers therefore need high-throughput radiomic features and multi-algorithm machine learning to comprehensively quantify the most stenotic level of the thoracic spinal canal, increasing the accuracy and clinical utility of outcome predictions on a larger scale. This study addresses that gap by using radiomic features from T2 axial MRI to build an integrated prediction model. Given the single-center and retrospective design, our work should be regarded as a preliminary exploratory analysis, providing early evidence to support individualized risk stratification and surgical decision-making in thoracic spinal stenosis.

## Methods

### Study population

From January 2012 to April 2022, 106 patients (49 men and 57 women) undergo surgical treatment at our hospital. Inclusion criteria are: (a) a clinical diagnosis of thoracic spinal canal stenosis with surgery led by a senior orthopedic surgeon; (b) availability of preoperative MRI; (c) high-quality images without motion artifacts; and (d) preoperative and long-term (≥3 years) follow-up modified Japanese Orthopedic Association (mJOA) scores. (e) Standardized posterior thoracic laminectomy with instrumentation is performed by a single senior spine surgeon. Exclusion criteria include (a) a history of thoracic surgery and (b) a history of other diseases (spinal cord tumor, multiple sclerosis, spinal cord sclerosis, spinal cord injury, or motor neuron disease). We collect clinical data on age, sex, and duration of symptoms. We assess neurological impairment using the JOA. Participants divide into a poor-outcome group (postoperative JOA < 16) and a good-outcome group (postoperative JOA ≥ 16), because a postoperative JOA under 16 still indicates severe residual deficits (11, 12).

### Extraction of MRI parameters

T2-weighted intramedullary high signal intensity (ISI) usually reflects intramedullary abnormalities from spinal cord compression. We evaluate it both qualitatively and quantitatively. ISI severity is classified into three levels: 0 for no signal change, 1 for mild and fuzzy high signal, and 2 for obvious and easily discernible bright signal (13, 14). In this study, we group ISI presence or absence into two categories due to sample-size considerations.

We use the spinal cord compression ratio to quantify how flattened the spinal cord appears in the compressed segment. The standard definition is: Spinal cord compression ratio = Minimum sagittal (Anterior–Posterior) diameter of the spinal cord at the compressed segment/Maximum transverse (Left to Right) diameter. A smaller ratio indicates a more flattened cord.

### Image preprocessing

All patients undergo MRI on a 3 T scanner in the head-to-supine position. We apply a standardized MRI preprocessing pipeline to reduce inter-image variability: (1) Resample images to ensure consistent resolution (2). Pre-crop the images around the spinal cord centerline to maintain uniform dimensions (3). Normalize image intensities to keep identical tissue types at consistent intensities. SCT (version 4.0.0)^1^ is applied for above process.

### ROI segmentation

On axial T2 images, we identify the most severely stenotic level, selecting that slice and adjacent slices as the region of interest (ROI). Because the intramedullary lesion (ISI) area is often small with unclear boundaries, we choose the entire compressed spinal cord cross-section as the ROI. Under the supervision of a senior spine surgeon, two independent spine surgeons verify the level. We use the intraclass correlation coefficient (ICC) to assess intra- and inter-observer reliability. Initially, one investigator delineates the ROI. Another investigator with over 10 years of neurosurgical experience then randomly selects 30 cases to independently re-delineate, both investigators remaining blinded to each other’s results. Using these 30 cases, we calculate ICC to measure consistency. We retain only radiomic features with ICC above 0.9 in both datasets for further analysis.

### Radiomics feature extraction

We carry out feature extraction using the Pyradiomics module.^2^ We enhance the range of derived images using filters such as the Laplacian of Gaussian and wavelets. All radiomics features fall into seven categories: shape-based features, first-order features, gray-level dependence matrix (GLDM) features, gray-level size zone matrix (GLSZM) features, neighboring gray-tone difference matrix (NGTDM) features, gray-level run-length matrix (GLRLM) features, and gray-level co-occurrence matrix (GLCM) features. The radiomics features are uploaded in Supplementary material 1.

### Feature selection

We use a rigorous approach to identify features most pertinent to thoracic spinal cord injury. First, a U-test (*p* < 0.05) pinpoints features with significant differences between the spinal cord injury group and the spinal cord concussion group. We exclude features with ICC under 0.9 at this stage. This strategy trims the number of features while preserving predictive power.

To address multicollinearity, we perform Spearman correlation analysis, examining inter-feature correlations. We label feature pairs with a correlation coefficient ≥0.9 or ≤ − 0.9 as strongly correlated and retain only the feature with superior diagnostic performance. Next, we use the minimum redundancy maximum relevance (mRMR) method to select the top 20 most important features. Finally, we apply the least absolute shrinkage and selection operator (LASSO) logistic regression to refine the feature set, imposing a penalty coefficient during variable selection and arriving at a more robust subset.

### Model construction

We use multiple machine learning algorithms (Random Forest, Bayesian, Neural Network, Decision Tree, Generalized Linear Model, and Support Vector Machine) on the selected radiomics features, employing SMOTE to balance the classes. To evaluate each classifier’s performance, we generate receiver operating characteristic (ROC) curves and calculate the area under the curve (AUC).

### Statistical analysis

We perform all statistical analyses using Python-based libraries (NumPy, Pandas, and SciPy, etc.). We use the AUC to measure predictive model performance. We employ DeLong’s test for comparing AUCs among different models to evaluate statistical differences in performance metrics. Analysis code and scripts are uploaded in Supplementary materials 2–4.

## Results

**Figure 1:**
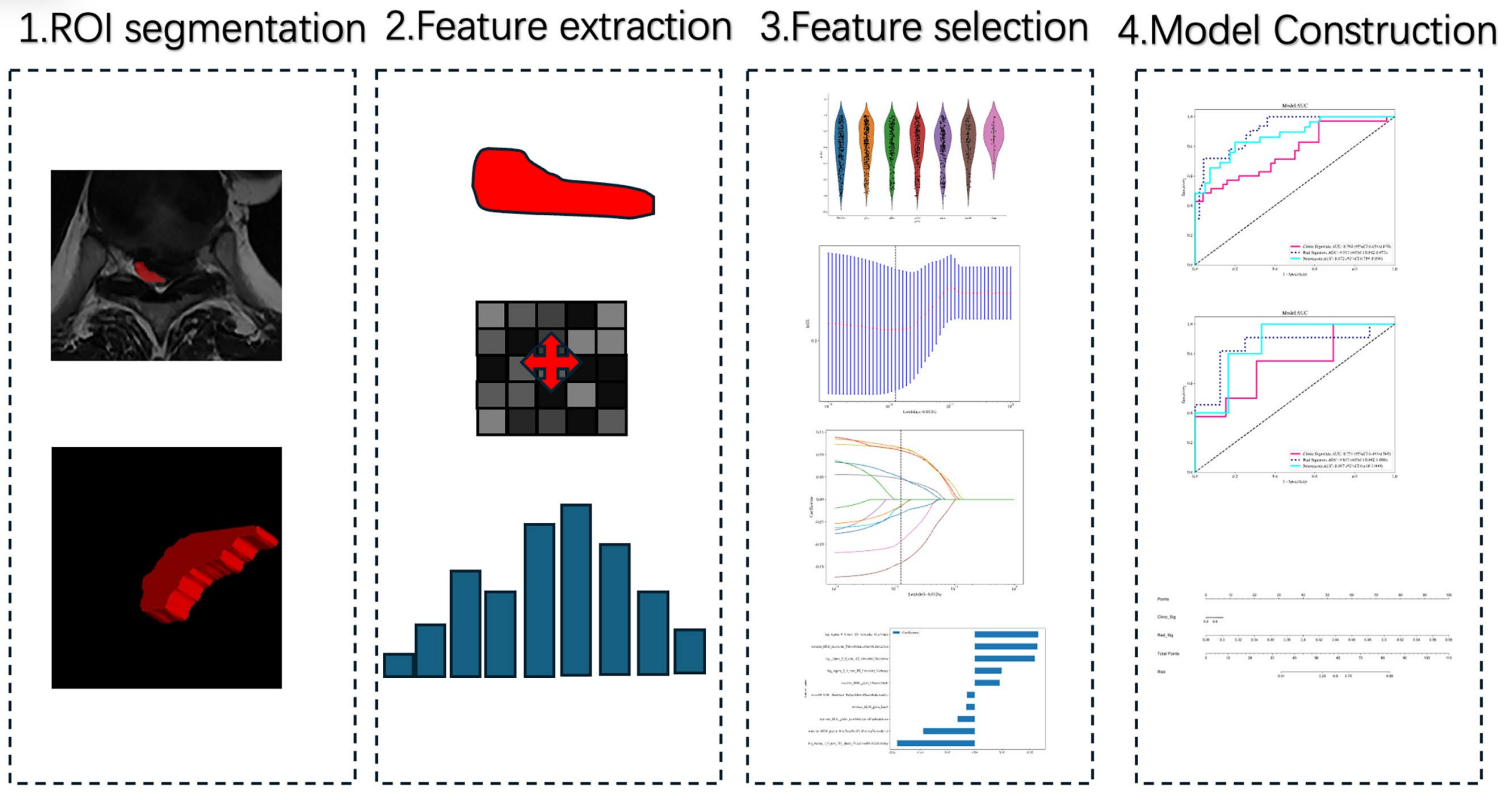
Workflow of radiomics analysis and model construction. Step 1: Region of interest (ROI) segmentation of the most stenotic thoracic spinal cord level on axial T2-weighted MRI. Step 2: Radiomics feature extraction from the segmented ROI. Step 3: Feature selection through reproducibility testing, statistical filtering, and LASSO regression. Step 4: Model construction using machine learning algorithms, followed by performance evaluation with ROC curves and nomogram visualization.

The study flowgram is shown in Figure 1. We include 106 patients with thoracic spinal canal stenosis in this study, dividing them into a Good outcome group (63 patients) and a Poor outcome group (43 patients). Their mean ages are 48.44 ± 10.39 years (Good) and 46.14 ± 10.89 years (Poor), with no statistically significant difference (*p* = 0.137) (Table 1). The sex ratio is similar in both groups, and the average follow-up durations are 45.17 ± 10.10 months and 43.32 ± 11.90 months, respectively, with no significant difference. The Poor group has a significantly longer symptom duration than the Good group (18.47 ± 6.70 vs. 13.84 ± 3.76 months, *p* < 0.001), suggesting that persistent symptoms may correlate with worse outcomes. On MRI, the Poor group shows a higher incidence of intramedullary high signal (ISI) (*p* = 0.04). Regarding preoperative neurological function, the Poor group’s baseline JOA score is distinctly lower than that of the Good group (6.88 ± 1.12 vs. 8.13 ± 1.67, *p* < 0.001). The Poor group also has a lower spinal cord compression ratio (0.1526 ± 0.0639 vs. 0.1951 ± 0.0621, *p* < 0.001), indicating more severe cord compression. At final follow-up, JOA scores differ significantly as well (11.38 ± 2.23 vs. 16.56 ± 0.50, *p* < 0.001).

**Table 1:** Patients’ demographics.

Variable	Train (*N* = 85)	Test (*N* = 21)	*P*
Age (year)	47.93 ± 10.65	45.81 ± 10.50	*p* = 0.207
Gender (male/female)	38/47	11/10	*p* = 0.53
Follow-up (m)	44.05 ± 10.40	45.95 ± 12.68	*p* = 0.237
JOA baseline	7.68 ± 1.60	7.38 ± 1.56	*p* = 0.219
ISI (yes/no)	41/44	13/8	*p* = 0.262
Compression rates	0.1791 ± 0.0644	0.1729 ± 0.0734	*p* = 0.351
Duration (m)	15.59 ± 5.57	16.24 ± 5.91	*p* = 0.319
JOA follow-up	14.61 ± 2.88	13.81 ± 3.17	*p* = 0.133
JOA outcomes (good/poor)	54/31	9/12	*p* = 0.08

We randomly assign all patients to a training set (85 cases) or a test set (21 cases) at about a 4:1 ratio. Baseline demographics and clinical characteristics do not significantly differ between sets (Table 2). Both sets show comparable clinical distributions and prognoses, meeting model development and validation needs.

**Table 2:** Patients’ demographics in train set and test set.

Variable	Good (*N* = 63)	Poor (*N* = 43)	*P*
Age	48.44 ± 10.39	46.14 ± 10.89	*P* = 0.137
Follow-up time (m)	45.17 ± 10.10	43.32 ± 11.9	*p* = 0.196
Gender (male/female)	27/36	22/21	*p* = 0.4
Duration (m)	13.84 ± 3.76	18.47 ± 6.70	*P* < 0.001
ISI (yes/no)	27/36	27/16	*P* = 0.04
JOA baseline	8.13 ± 1.67	6.88 ± 1.12	*P* < 0.001
Compression rate	0.1951 ± 0.0621	0.1526 ± 0.0639	*P* < 0.001
JOA follow-up	16.56 ± 0.5	11.38 ± 2.23	*P* < 0.001

### Clinical model construction

**Figure 2:**
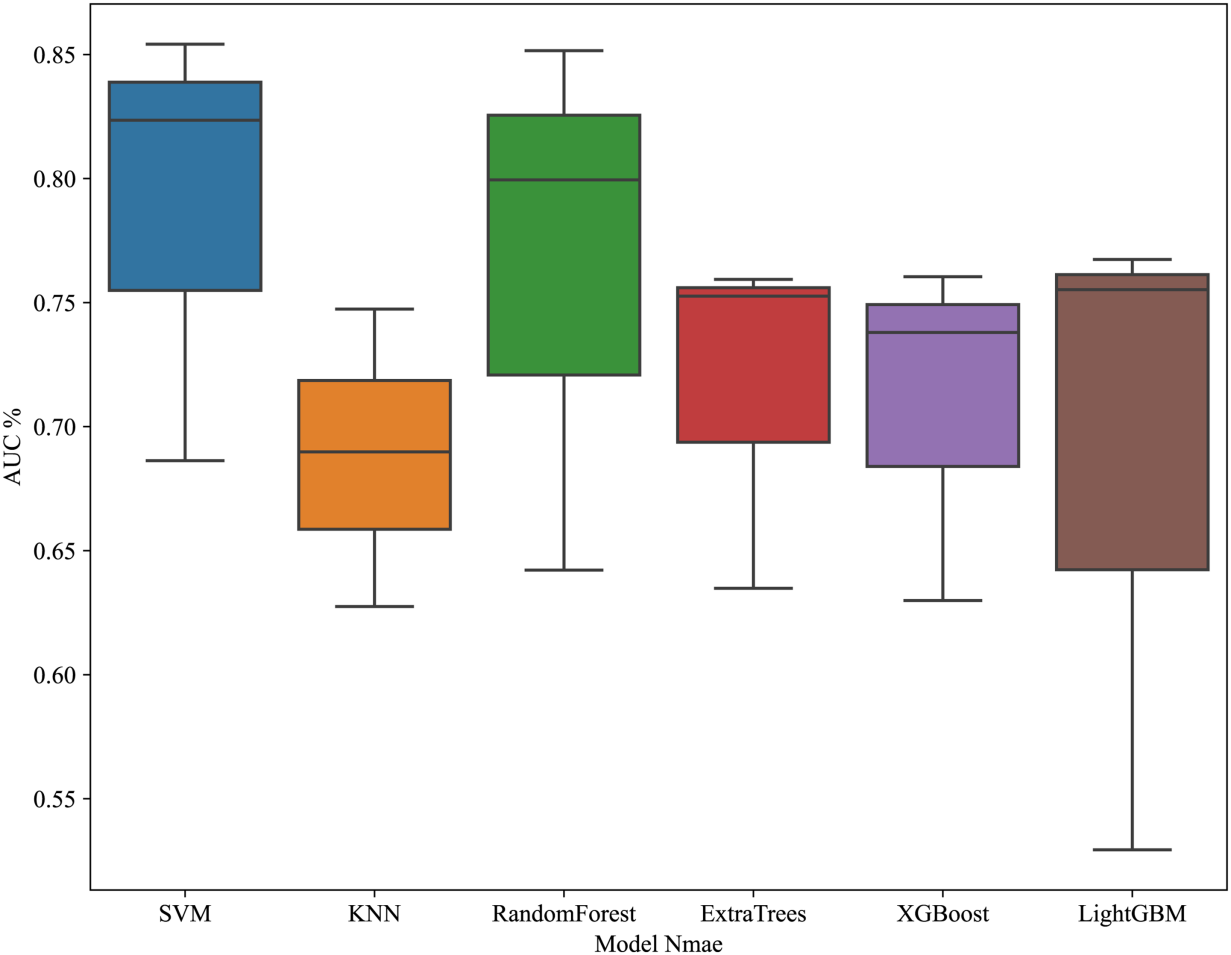
Cross-validation performance of the clinical model.

**Figure 3:**
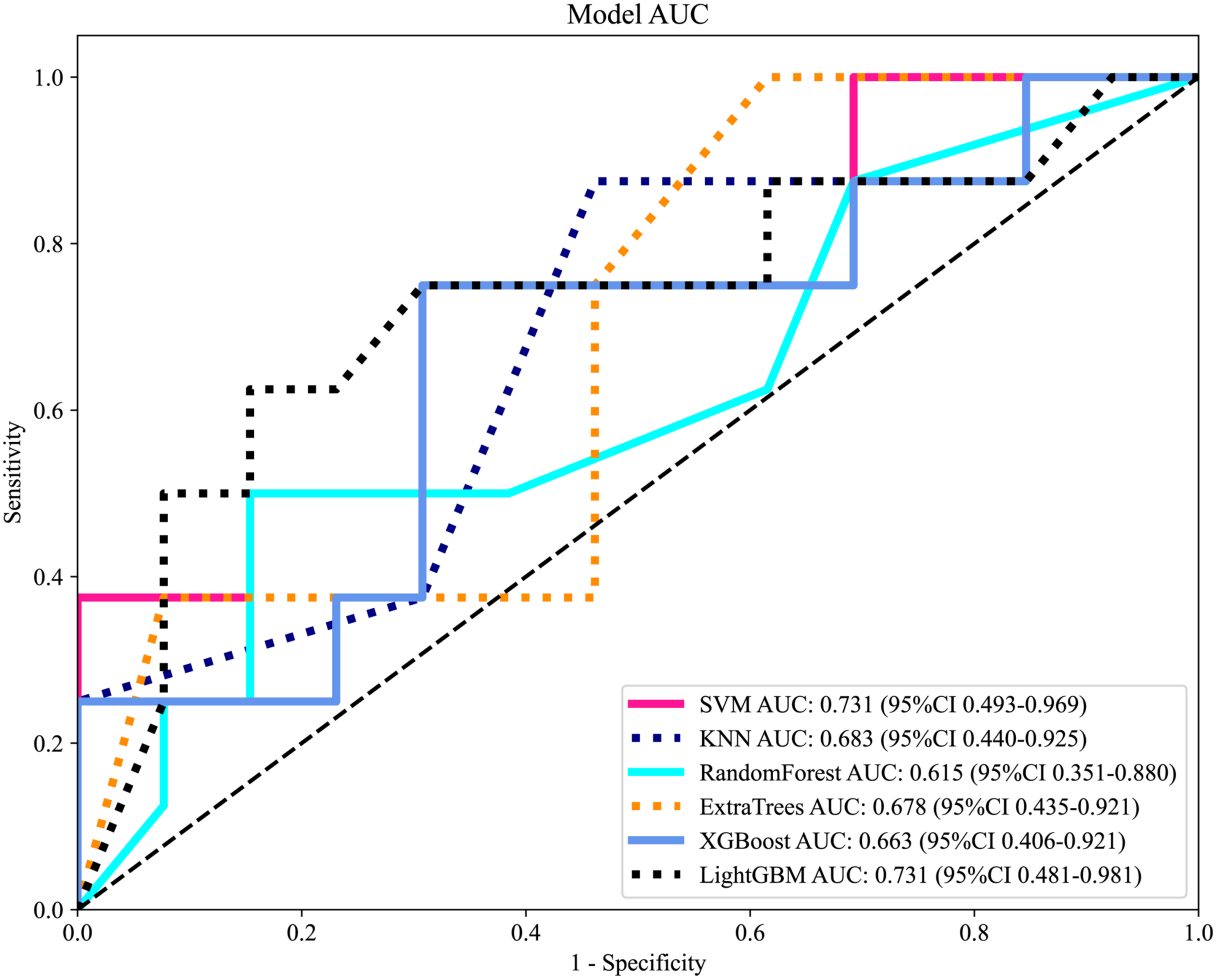
Receiver operating characteristic (ROC) curves of different machine learning classifiers based on clinical features in the independent test set. Models included Support Vector Machine (SVM), K-Nearest Neighbors (KNN), Random Forest, Extra Trees, XGBoost, and LightGBM.

JOA baseline, High intensity signal, duration and compression rates are applied in clinical model construction. The cross validation is shown in Figure 2. Table 3 summarizes different machine learning algorithm. The best clinical model achieves an AUC of 0.731 on the test set (SVM), with an accuracy of 66.7%, a sensitivity of 62.5%, and a specificity of 69.2%. Figure 3 shows the ROC curves and AUCs for each machine learning algorithm in the clinical model for the test set.

**Table 3:** Comparison of machine learning performance of clinical model.

	Model	Accuracy	AUC	Sensitivity	Specificity	PPV	NPV	Precision	Recall	F1	Threshold	Task
0	SVM	0.753	0.764	0.457	0.960	0.889	0.716	0.889	0.457	0.604	0.538	Label-train
1	SVM	0.667	0.731	0.625	0.692	0.556	0.750	0.556	0.625	0.588	0.286	Label-test
2	KNN	0.788	0.865	0.486	1.000	1.000	0.’ 735	1.000	0.486	0.654	0.600	Label-train
3	KNN	0.571	0.683	0.375	0.692	0.429	0.643	0.429	0.375	0.400	0.400	Label-test
4	RandomForest	0.965	0.998	0.914	1.000	1.000	0.943	1.000	0.914	0.955	0.500	Label-train
5	RandomForest	0.619	0.615	0.250	0.846	0.500	0.647	0.500	0.250	0.333	0.500	Label-test
6	ExtraTrees	0.588	1.000	0.000	1.000	0.000	0.588	0.000	0.000	NaN	1.000	Label-train
7	ExtraTrees	0.619	0.678	0.750	0.538	0.500	0.778	0.500	0.750	0.600	0.200	Label-test
8	XGBoost	0.918	0.978	0.943	0.900	0.868	0.957	0.868	0.943	0.904	0.335	Label-train
9	XGBoost	0.667	0.663	0.625	0.692	0.556	0.750	0.556	0.625	0.588	0.270	Label-test
10	LightGBM	0.776	0.834	0.657	0.860	0.767	0.782	0.767	0.657	0.708	0.397	Label-train
11	LightGBM	0.714	0.731	0.500	0.846	0.667	0.733	0.667	0.500	0.571	0.389	Label-test

### LASSO regression results

#### Radiomics feature selection and radiomics signature building

**Figure 4:**
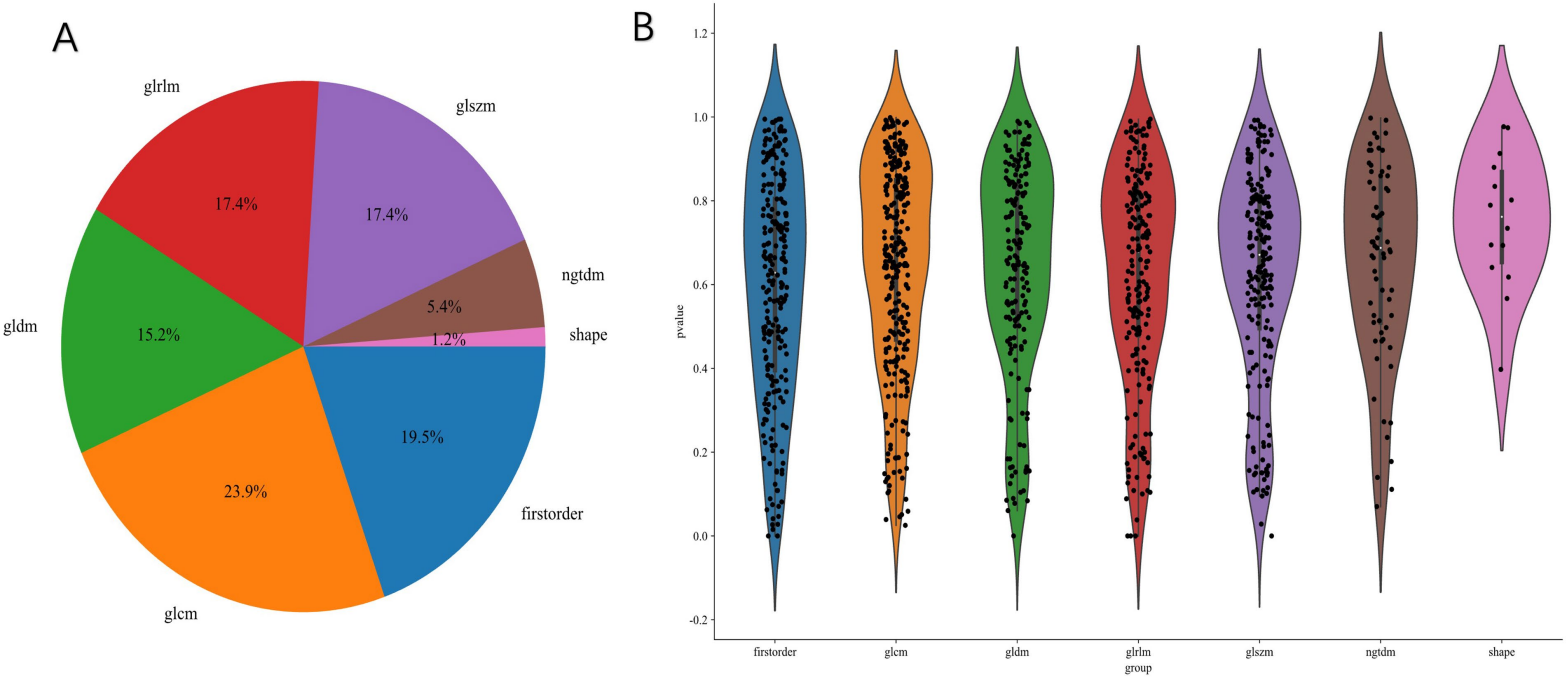
Radiomics feature extraction results. **(A)** Distribution of extracted features across seven categories (first-order, GLCM, GLDM, GLRLM, GLSZM, NGTDM, and shape). **(B)** Violin plots of p-values for different feature categories following univariate filtering, showing significant feature diversity across groups.

**Figure 5:**
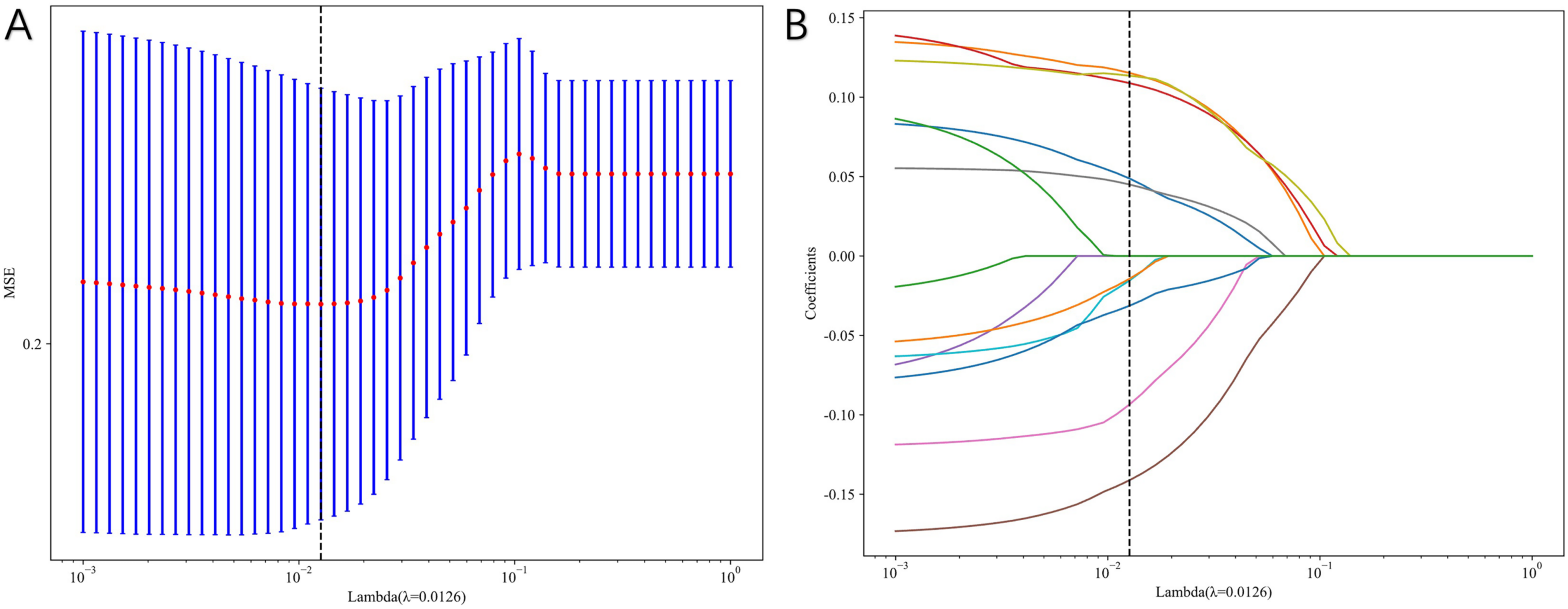
Radiomics feature selection using least absolute shrinkage and selection operator (LASSO) regression. **(A)** Ten-fold cross-validation plot used to determine the optimal λ. **(B)** Coefficient profiles of radiomics features, with 10 non-zero features retained for model construction.

**Figure 6:**
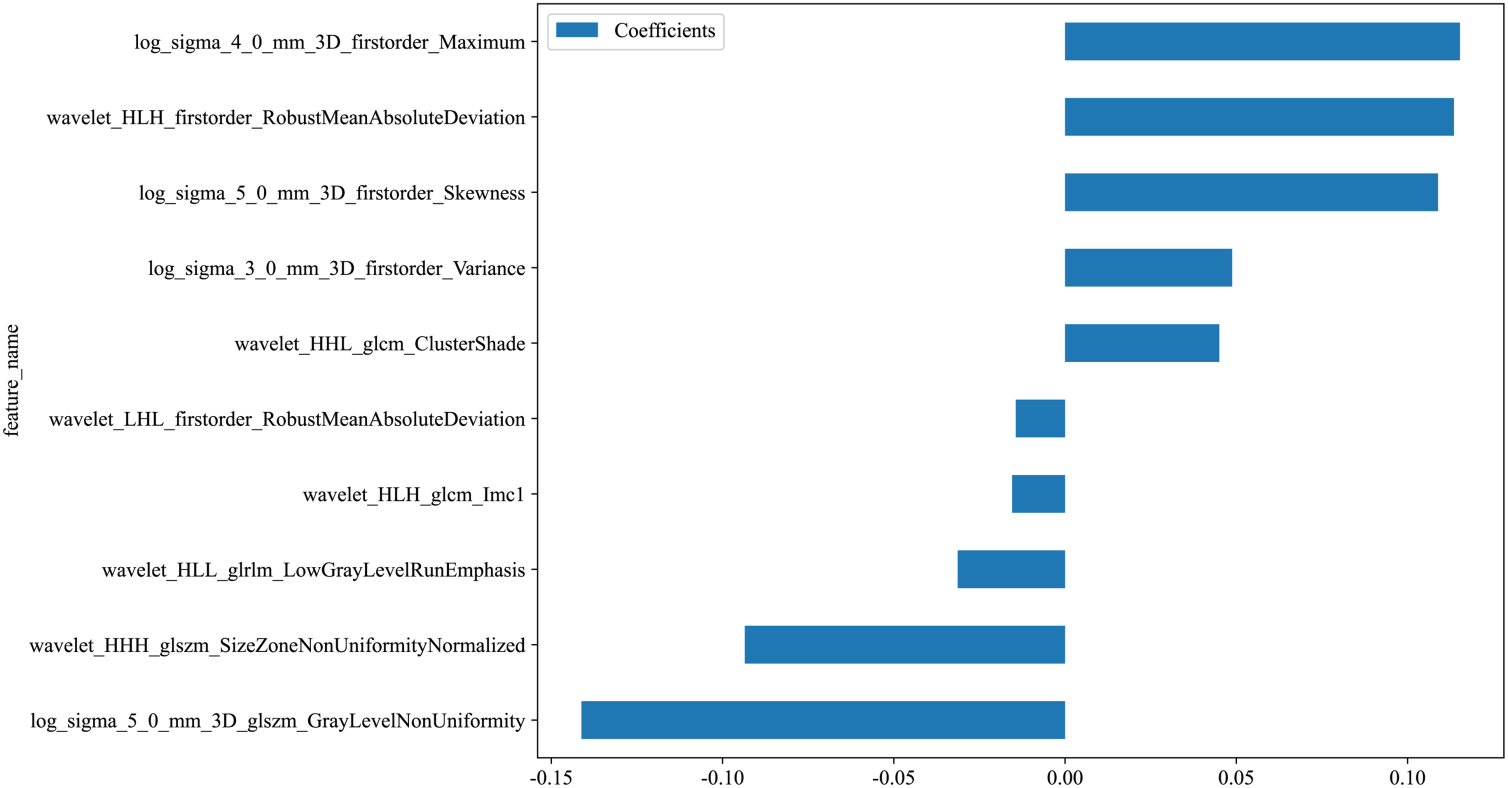
Weights of selected radiomics features after LASSO regression. Bar plots show the relative contributions of the 10 retained features to the final radiomics signature.

We extract a total of 1,198 radiomics features from thoracic stenosis MRI data, including 234 first-order features, 286 GLCM features, 182 GLDM features, 208 GLRLM features, 208 GLSZM features, 65 NGTDM features, and 14 shape features (shown in Figure 4). After validation, radiomics feature reproducibility is satisfactory. We use the LASSO algorithm in the training set to find the optimal regularization weight (*λ* = 0.0126), selecting 10 radiomics features that predict outcomes in thoracic spinal canal stenosis, shown in Figure 5. Figure 6 shows the coefficient distribution for these features. The radiomics score uses this formula:

label = 0.3090601683388831 + +0.127998 * original_firstorder_Kurtosis −0.077429 * original_firstorder_Minimum +0.114108 * original_firstorder_RootMeanSquared −0.047303 * original_glcm_Imc2–0.063017 * original_glcm_SumEntropy +0.064179 * original_glrlm_LongRunLowGrayLevelEmphasis −0.059840 * original_glrlm_RunEntropy −0.031554 * original_glrlm_ShortRunEmphasis −0.016628 * original_glszm_GrayLevelVariance +0.008884 * original_glszm_ZoneVariance +0.053577 * original_ngtdm_Busyness −0.029125 * original_shape_Sphericity

#### Model construction

**Figure 7:**
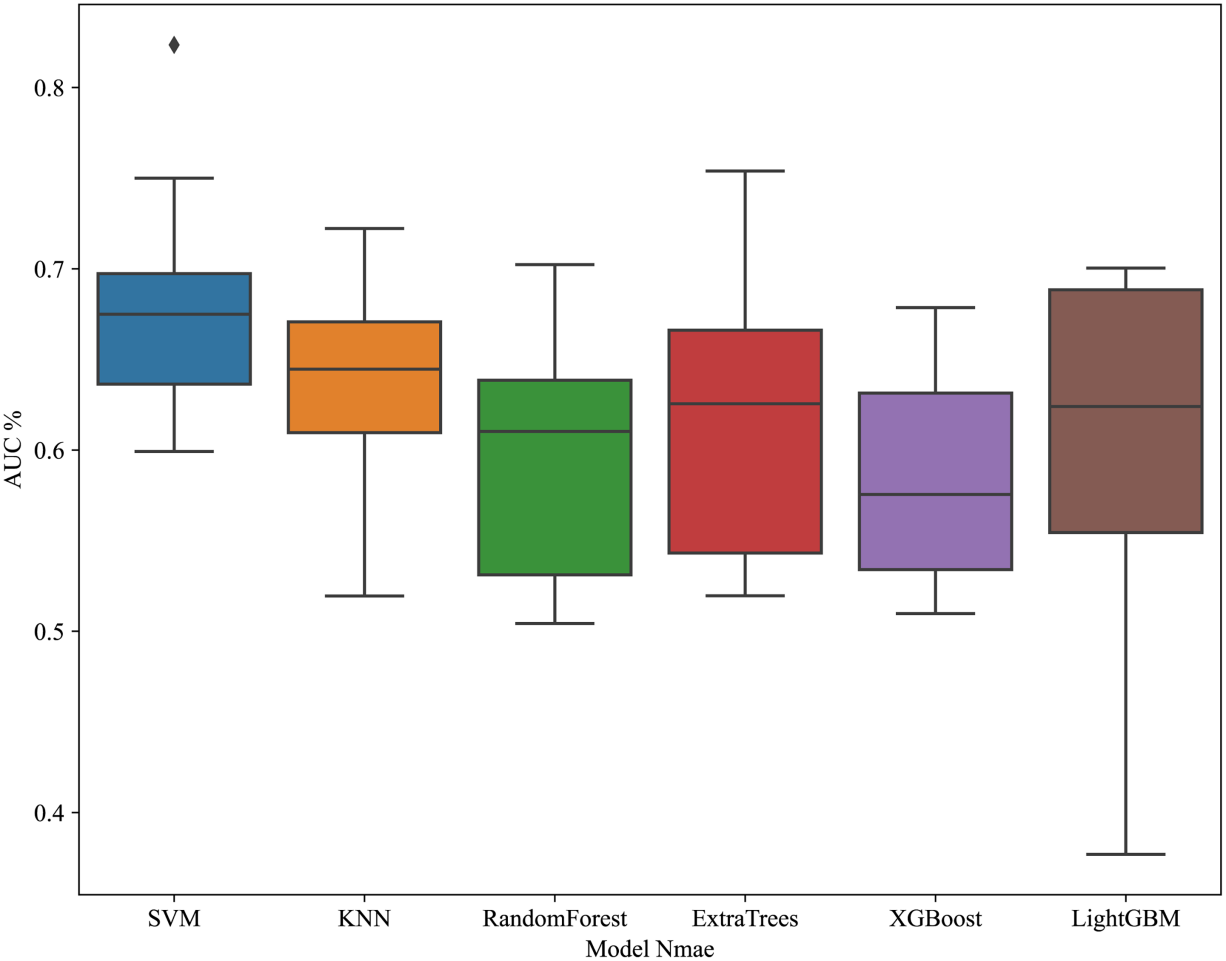
Cross-validation performance of radiomics-based models. ROC curves demonstrate performance of different machine learning classifiers in the training set using radiomics features only.

**Figure 8:**
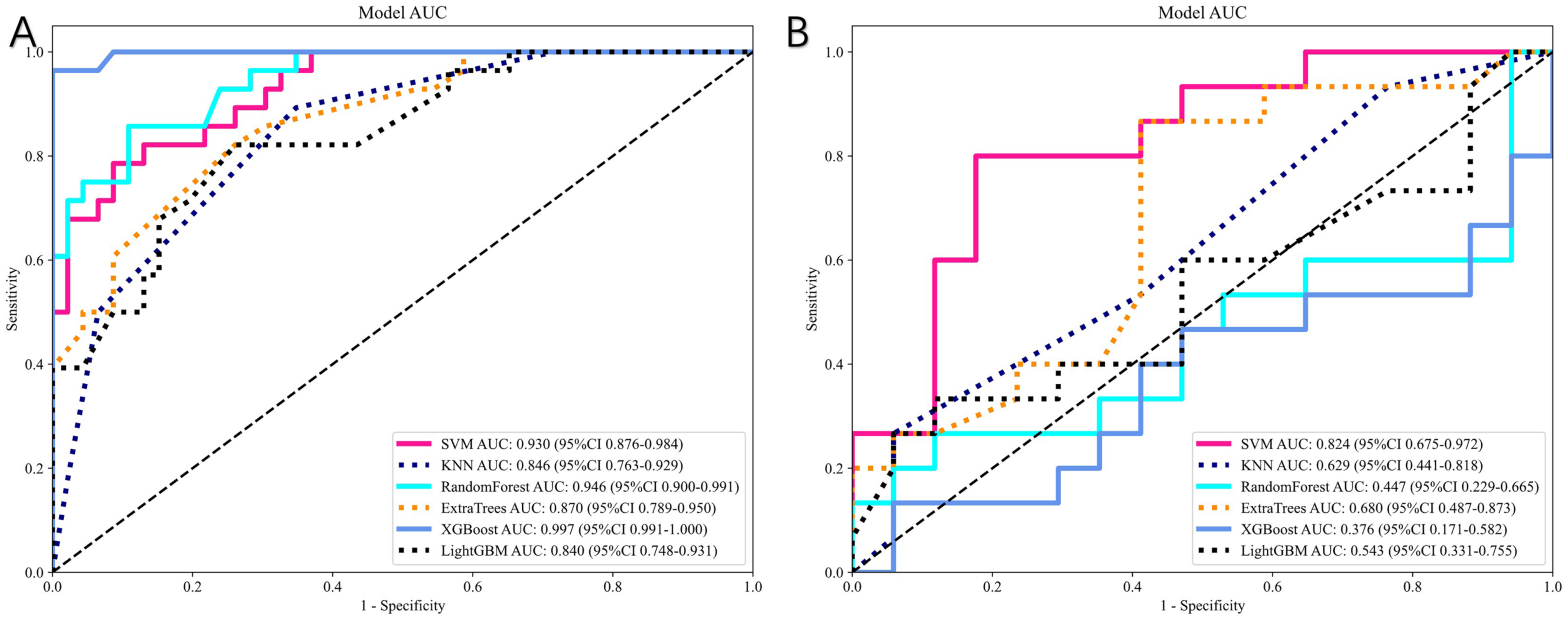
Performance of radiomics-based models. **(A)** ROC curves in the training cohort. **(B)** ROC curves in the independent test cohort. The SVM-based radiomics model achieved the best predictive performance (test-set AUC = 0.824).

We build models using various machine learning algorithms based on radiomics features alone, then compare their performance (Table 4). The cross validation result is shown in Figure 7. Among radiomics-based models, SVM demonstrates the best test-set performance, with an AUC of 0.824, an accuracy of 78.1%, a sensitivity of 73.3%, and a specificity of 82.4%. Figure 8 shows the radiomics model’s ROC curves and AUCs in the training set (A) and test set (B).

**Table 4:** Comparison of machine learning performance of radiomics model.

	Model_	Accuracy	AUC	Sensitivity	Specificity	PPV	NPV	Precision	Recall	F1	Threshold	Task
0	SVM	0.851	0.930	0.750	0.913	0.840	0.857	0.840	0.750	0.792	0.378	Label-train
1	SVM	0.781	0.824	0.733	0.824	0.786	0.778	0.786	0.733	0.759	0.351	Label-test
2	KNN	0.7 70	0.846	0.500	0.935	0.824	0.’ 754	0.824	0.500	0.622	0.400	Label-train
3	KNN	0.531	0.629	0.067	0.941	0.500	0.533	0.500	0.067	0.118	0.600	Label-test
4	RandomForest	0.865	0.946	0.821	0.891	0.821	0.891	0.821	0.821	0.821	0.412	Label-train
5	RandomForest	0.562	0.447	0.200	0.882	0.600	0.556	0.600	0.200	0.300	0.540	Label-test
6	ExtraTrees	0.797	0.870	0.607	0.913	0.810	0.792	0.810	0.607	0.694	0.394	Label-train
7	ExtraTrees	0.688	0.680	0.800	0.588	0.632	0.’ 769	0.632	0.800	0.’ 706	0.352	Label-test
8	XGBoost	0.973	0.997	0.929	1.000	1.000	0.958	1.000	0.929	0.963	0.437	Label-train
9	XGBoost	0.531	0.376	0.067	0.941	0.500	0.533	0.500	0.067	0.118	0.711	Label-test
10	LightGBM	0.770	0.840	0.786	0.761	0.667	0.854	0.667	0.786	0.721	0.355	Label-train
11	LightGBM	0.594	0.543	0.267	0.882	0.667	0.577	0.667	0.267	0.381	0.481	Label-test

#### Radiomics-clinical model

**Figure 9:**
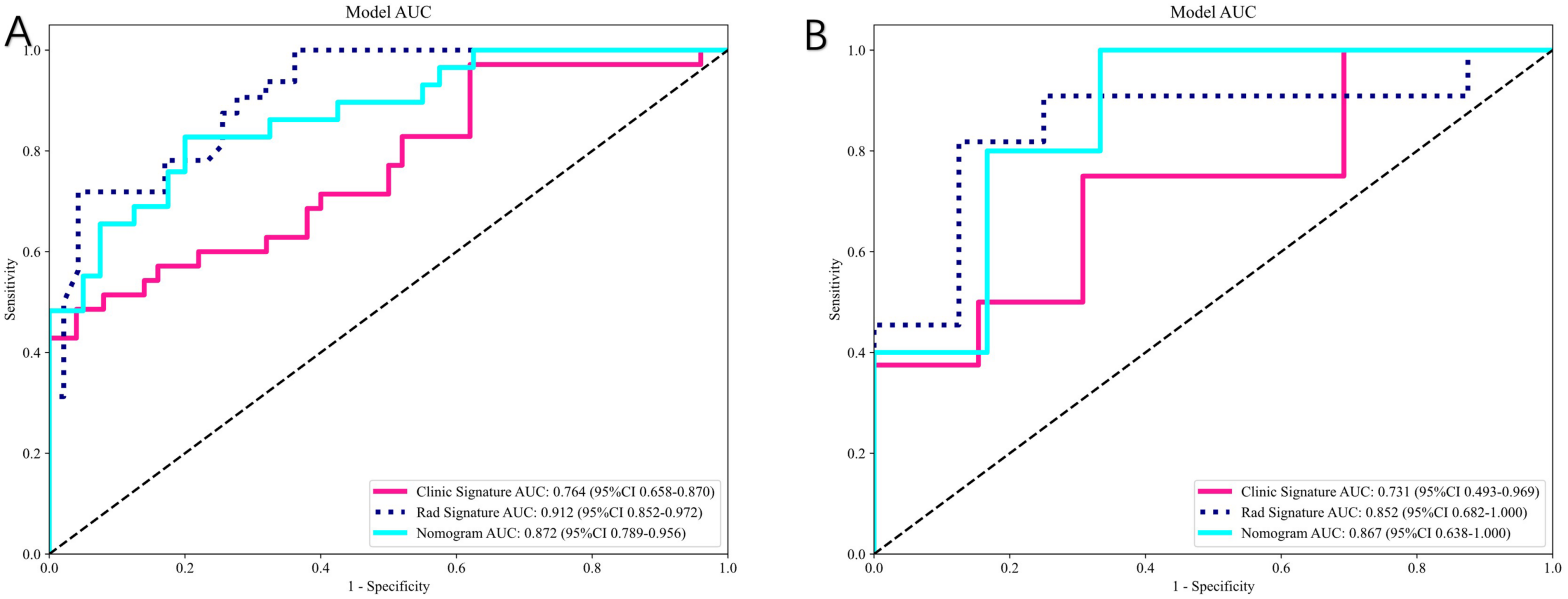
Comparison of clinical, radiomics, and combined (nomogram) models. **(A)** ROC curves in the training set. **(B)** ROC curves in the test set. The combined model integrating radiomics and clinical features achieved the best overall predictive performance (test-set AUC = 0.867).

**Figure 10:**
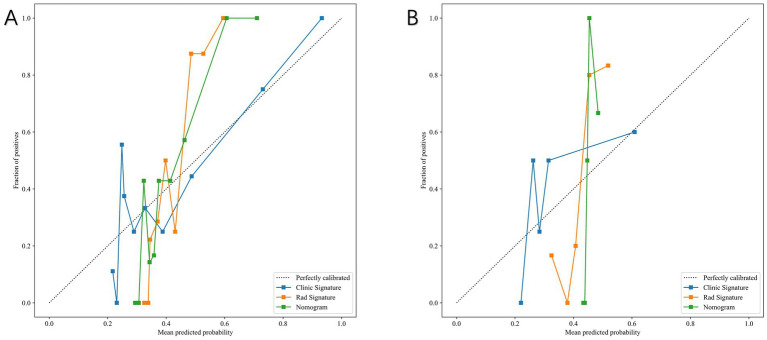
Calibration curves of the clinical model, radiomics model, and combined nomogram model. **(A)** Training set. **(B)** Test set. The combined model demonstrated the best agreement between predicted and observed outcomes.

**Figure 11:**
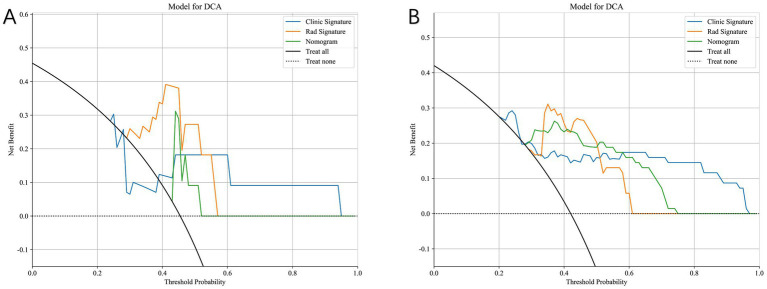
Decision curve analysis (DCA) of the clinical model, radiomics model, and combined nomogram model. **(A)** Training set. **(B)** Test set. The nomogram consistently provides a higher net clinical benefit across a wider range of threshold probabilities compared with models using clinical or radiomics features alone.

Figure 9 compares the ROC curves of the clinical model, the radiomics model, and the combined model in both the training set (A) and test set (B). Each model uses its best-performing algorithm, and the combined model merges them into a nomogram. Table 5 summarizes the clinical, radiomics, and combined models. In the training set, the radiomics model yields the highest AUC and significantly exceeds the clinical model, while the combined model reaches an AUC of 0.872. In the test set, the combined model’s ROC curve shows an AUC of 0.867, representing the best performance overall. Figure 10 shows Calibration curves between three models in train set (A) and test set (B). And Figure 11 shows DCA curves between three models in train set (A) and test set (B).

**Table 5:** Comparative performance of clinical, radiomics, and combined models.

Model-name	Accuracy	AUC	Sensitivity	Specificity	PPV	NPV	Precision	Recall	F1	Threshold	Task
Clinic signature	0.737	0.783	0.792	0.712	0.559	0.881	0.559	0.792	0.655	0.306	Train
Rad signature	0.750	0.835	0.750	0.750	0.581	0.867	0.581	0.750	0.655	0.319	Train
Nomogram	0.816	0.902	0.875	0.788	0.656	0.932	0.656	0.875	0.750	0.322	Train
Clinic signature	0.750	0.708	0.333	0.929	0.667	0.’ 765	0.667	0.333	0.444	0.378	Test
Rad signature	0.700	0.845	0.667	0.714	0.500	0.833	0.500	0.667	0.571	0.319	Test
Nomogram	0.700	0.857	0.833	0.643	0.500	0.900	0.500	0.833	0.625	0.298	Test

**Figure 12:**
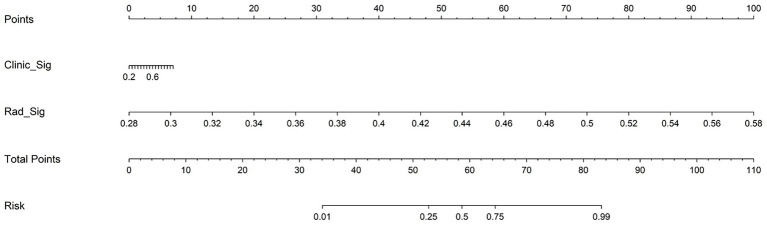
Nomogram integrating radiomics signature and clinical signature for predicting postoperative neurological recovery in thoracic spinal stenosis. The total points are calculated by summing the scores for clinical and radiomics predictors, which correspond to the estimated probability of poor outcome.

Figure 12 presents the nomogram based on radiomics plus clinical features, integrating both elements to predict individual surgical outcomes. The above ROC analysis supports its effectiveness (Table 6).

**Table 6:** Delong test of three models.

Results	Nomogram vs. clinic	Nomogram vs. rad	Cohort
Good	0.003	0.308	Train
Poor	0.001	0.009	Test

## Discussion

This study constructs multiple machine learning models using T2 axial MRI radiomics features and clinical variables to predict neurological recovery after surgery in thoracic spinal stenosis. The combined model (radiomics + clinical) provides the best predictive power, achieving an AUC of 0.867 in the test set, surpassing models that include only clinical or only imaging data. This finding indicates that incorporating high-dimensional MRI quantitative features with patient clinical information greatly enhances the ability to discriminate between good and poor postoperative neurological recovery, outperforming traditional empirical assessments.

In recent years, multiple studies in fields such as cervical spine pathologies or spinal cord injuries verify that radiomics holds promise for outcome prediction, treatment evaluation, and individualized decision-making (10, 15–17). Consistent with those findings, our study shows that radiomics-based modeling outperforms models relying solely on clinical factors, and that merging radiomics and clinical data further boosts the predictive capacity for postoperative neurological outcomes.

Clinically, TSS prognosis usually depends on surgeon experience and a few specific factors—such as preoperative symptom severity or MRI findings—but these single-factor predictions have limited accuracy (2). For instance, T2 intramedullary high signal is often regarded as a marker of severe cord damage and an indicator for poor outcome, but its predictive power varies across studies. Kozaki and Yukawa report higher intensity is associated with worse outcomes (18, 19). But traditional MRI qualitative indicators fail to capture the complete complexity of lesion properties, leading to suboptimal preoperative risk stratification.

Our findings reveal that clinical factors alone (e.g., symptom duration, preoperative JOA score) offer limited predictive accuracy for TSS outcomes, whereas high-throughput radiomic features from MRI markedly enhance model discrimination. Radiomics extracts a multitude of objective texture, shape, and grayscale distribution features from standard MRI, capturing finer lesion details and spinal cord heterogeneity that are imperceptible to the naked eye. These high-dimensional, quantitative variables characterize intramedullary changes and cord deformation more comprehensively than single metrics like T2 high signal presence or maximum compression ratio. We observe that a radiomics-based model raises the AUC to around 0.74 or higher, and that incorporating clinical data further boosts performance to 0.867, significantly surpassing any single-factor approach. This result implies that a multimodal model can detect the complex combination of factors influencing outcomes, thereby providing better predictive power than existing methods.

Radiomics also shows clinical potential by offering objective spinal cord injury assessment and individualized estimates of surgical benefit. Subtle imaging differences often reflect various pathological processes, such as intramedullary degeneration, inflammatory edema, microhemorrhages, or local blood supply changes, which single imaging signs or subjective observations frequently miss. By modeling high-dimensional radiomics, clinicians can better quantify the interplay among these pathological factors, identify high-risk patients preoperatively, and optimize surgical timing and approach.

Moreover, radiomics easily integrates with artificial intelligence algorithms, allowing for the creation of comprehensive decision-support systems that merge imaging, clinical characteristics, and surgical parameters. Compared to traditional regression models, machine learning (e.g., random forests, SVMs, and neural networks) excels at handling complex, nonlinear data, enabling more precise, individualized prognosis predictions for TSS patients. This is especially valuable for a patient population prone to wide variability in postoperative functional recovery and in need of timely interventions.

Compared with existing prediction methods, our combined radiomics-based model offers multiple advantages and strong clinical feasibility. First, radiomics analysis objectively extracts numerous MRI features, reducing subjective bias and capturing subtle imaging details relevant to spinal canal morphology, spinal cord compression, and signal heterogeneity. Second, machine learning algorithms incorporate this multidimensional information and uncover nonlinear relationships between imaging biomarkers and clinical data, improving predictive accuracy. Our findings confirm that a multi-factor model outperforms any single-factor approach, highlighting the potential of statistical learning in complex clinical prediction tasks.

Nonetheless, this study faces certain limitations:(1) Study Design: This is a single-center retrospective study with a relatively small sample size, which may limit model robustness and generalizability. Larger datasets from multiple centers and regions would strengthen external validation. Prospective, multicenter designs also help control confounders and further validate clinical applicability. (2) ROI Segmentation: We manually delineate the lesion region, which introduces observer subjectivity. Although it ensures some accuracy, operator variability still exists. Future studies may adopt semi-automated or fully automated computer-assisted segmentation tools to reduce manual bias.

## Conclusion

This preliminary study suggests that integrating T2 axial MRI radiomics with clinical variables via machine learning may enhance the prediction of postoperative neurological recovery in thoracic spinal stenosis. While promising, these findings remain exploratory and require external validation in larger, prospective, multicenter studies.

## Data Availability

The original contributions presented in the study are included in the article/Supplementary material, further inquiries can be directed to the corresponding author.

## Footnotes

^1^ https://github.com/neuropoly/spinalcordtoolbox

^2^ https://github.com/Radiomics/pyradiomics

## References

[1] Chen G Fan T Yang X Sun C Fan D Chen Z. The prevalence and clinical characteristics of thoracic spinal stenosis: a systematic review. Eur Spine J. (2020) 29:2164–72. doi: 10.1007/s00586-020-06520-6, PMID: 32671614

[2] Zhang J Wang L Li J Yang P Shen Y. Predictors of surgical outcome in thoracic ossification of the ligamentum flavum: focusing on the quantitative signal intensity. Sci Rep. (2016) 6:23019. doi: 10.1038/srep23019, PMID: 26960572 PMC4785339

[3] Kim TH Ha Y Shin JJ Cho YE Lee JH Cho WH. Signal intensity ratio on magnetic resonance imaging as a prognostic factor in patients with cervical compressive myelopathy. Medicine. (2016) 95:e4649. doi: 10.1097/MD.0000000000004649, PMID: 27684796 PMC5265889

[4] Hitchon PW Abode-Iyamah K Dahdaleh NS Grossbach AJ el Tecle NE Noeller J. Risk factors and outcomes in thoracic stenosis with myelopathy: a single center experience. Clin Neurol Neurosurg. (2016) 147:84–9. doi: 10.1016/j.clineuro.2016.05.029, PMID: 27310291

[5] Alsoof D McDonald CL Durand WM Diebo BG Kuris EO Daniels AH. Radiomics in spine surgery. Int J Spine Surg. (2023) 17:S57–s64. doi: 10.14444/8501 37193607 PMC10318908

[6] Cheng L Cai F Xu M Liu P Liao J Zong S. A diagnostic approach integrated multimodal radiomics with machine learning models based on lumbar spine CT and X-ray for osteoporosis. J Bone Miner Metab. (2023) 41:877–89. doi: 10.1007/s00774-023-01469-0, PMID: 37898574

[7] Gitto S Bologna M Corino VDA Emili I Albano D Messina C. Diffusion-weighted MRI radiomics of spine bone tumors: feature stability and machine learning-based classification performance. Radiol Med. (2022) 127:518–25. doi: 10.1007/s11547-022-01468-7, PMID: 35320464 PMC9098537

[8] Li S Yu X Shi R Zhu B Zhang R Kang B. MRI-based radiomics nomogram for differentiation of solitary metastasis and solitary primary tumor in the spine. BMC Med Imaging. (2023) 23:29. doi: 10.1186/s12880-023-00978-8, PMID: 36755233 PMC9909949

[9] Saravi B Zink A Ülkümen S Couillard-Despres S Wollborn J Lang G. Clinical and radiomics feature-based outcome analysis in lumbar disc herniation surgery. BMC Musculoskelet Disord. (2023) 24:791. doi: 10.1186/s12891-023-06911-y, PMID: 37803313 PMC10557221

[10] Lin F Wang K Lai M Wu Y Chen C Wang Y. Multicenter study on predicting postoperative upper limb muscle strength improvement in cervical spinal cord injury patients using radiomics and deep learning. Sci Rep. (2025) 15:5805. doi: 10.1038/s41598-024-72539-0, PMID: 39962172 PMC11833087

[11] Tetreault LA Kopjar B Vaccaro A Yoon ST Arnold PM Massicotte EM. A clinical prediction model to determine outcomes in patients with cervical spondylotic myelopathy undergoing surgical treatment: data from the prospective, multi-center AOSpine North America study. J Bone Joint Surg Am. (2013) 95:1659–66. doi: 10.2106/JBJS.L.01323, PMID: 24048553

[12] Nouri A Tetreault L Côté P Zamorano JJ Dalzell K Fehlings MG. Does magnetic resonance imaging improve the predictive performance of a validated clinical prediction rule developed to evaluate surgical outcome in patients with degenerative cervical myelopathy? Spine. (2015) 40:1092–100. doi: 10.1097/BRS.0000000000000919, PMID: 25893357

[13] Machino M Imagama S Ando K Kobayashi K Ito K Tsushima M. Image diagnostic classification of magnetic resonance T2 increased signal intensity in cervical spondylotic myelopathy: clinical evaluation using quantitative and objective assessment. Spine. (2018) 43:420–6. doi: 10.1097/BRS.0000000000002328, PMID: 28704332

[14] Wei L Wei Y Tian Y Cao P Yuan W. Does three-grade classification of T2-weighted increased signal intensity reflect the severity of myelopathy and surgical outcomes in patients with cervical compressive myelopathy? A systematic review and meta-analysis. Neurosurg Rev. (2020) 43:967–76. doi: 10.1007/s10143-019-01106-3, PMID: 31053986

[15] Zhang MZ Ou-Yang HQ Jiang L Wang CJ Liu JF Jin D. Optimal machine learning methods for radiomic prediction models: clinical application for preoperative T(2)*-weighted images of cervical spondylotic myelopathy. JOR Spine. (2021) 4:e1178. doi: 10.1002/jsp2.1178, PMID: 35005444 PMC8717093

[16] Zhang MZ Ou-Yang HQ Liu JF Jin D Wang C-J Ni M. Predicting postoperative recovery in cervical spondylotic myelopathy: construction and interpretation of T(2)(*)-weighted radiomic-based extra trees models. Eur Radiol. (2022) 32:3565–75. doi: 10.1007/s00330-021-08383-x, PMID: 35024949

[17] Zhang Z Li N Ding Y Cheng H. An integrative nomogram based on MRI radiomics and clinical characteristics for prognosis prediction in cervical spinal cord injury. Eur Spine J. (2025) 34:1164–76. doi: 10.1007/s00586-024-08609-8, PMID: 39672993

[18] Kozaki T Yukawa Y Hashizume H Iwasaki H Tsutsui S Takami M. Clinical and radiographic characteristics of increased signal intensity of the spinal cord at the vertebral body level in patients with cervical myelopathy. J Orthop Sci. (2023) 28:1240–5. doi: 10.1016/j.jos.2022.10.010, PMID: 36396505

[19] Yukawa Y Kato F Yoshihara H Yanase M Ito K. MR T2 image classification in cervical compression myelopathy: predictor of surgical outcomes. Spine. (2007) 32:1675–8. doi: 10.1097/BRS.0b013e318074d62e 17621217

